# Intravenously Injected Mesenchymal Stem Cells Penetrate the Brain and Treat Inflammation-Induced Brain Damage and Memory Impairment in Mice

**DOI:** 10.3389/fphar.2019.00355

**Published:** 2019-04-17

**Authors:** Olena Lykhmus, Lyudmyla Koval, Larysa Voytenko, Kateryna Uspenska, Serhiy Komisarenko, Olena Deryabina, Nadia Shuvalova, Vitalii Kordium, Alina Ustymenko, Vitalii Kyryk, Maryna Skok

**Affiliations:** ^1^ Laboratory of Cell Receptors Immunology, Palladin Institute of Biochemistry NAS, Kyiv, Ukraine; ^2^ Department of Gene Technologies, State Institute of Genetic and Regenerative Medicine NAMS, Kyiv, Ukraine; ^3^ Department of Cell Regulatory Mechanisms, Institute of Molecular Biology and Genetics NAS, Kyiv, Ukraine

**Keywords:** neuroinflammation, mesenchymal stem cells, nicotinic acetylcholine receptor, episodic memory, mitochondria, Alzheimer disease

## Abstract

Neuroinflammation is regarded as one of the pathogenic factors of Alzheimer disease (AD). Previously, we showed that mice regularly injected with bacterial lipopolysaccharide (LPS) possessed the AD-like symptoms like episodic memory decline, elevated amounts of amyloid beta (Aβ) peptide (1–42), and decreased levels of nicotinic acetylcholine receptors (nAChRs) in the brain. The use of mesenchymal stem cells (MSCs), which can differentiate into multiple cell types, including neurons, is an attractive idea of regenerative medicine, in particular, for neurodegenerative disorders like AD. In the present study, we aimed to investigate whether pathogenic effect of LPS on the brain and behavior of mice can be prevented or treated by injection of MSCs or MSC-produced soluble factors. Fluorescently-labeled MSCs, injected intravenously, were found in the brain blood vessels of LPS-treated mice. Mice co-injected with LPS and MSCs did not demonstrate episodic memory impairment, Aβ (1–42) accumulation, and nAChR decrease in the brain and brain mitochondria. Their mitochondria released less cytochrome *c* under the effect of Ca^2+^ compared to mitochondria of LPS-only-treated mice. Moreover, MSCs could reverse the pathogenic symptoms developed 3 weeks after LPS injection. Cultured MSCs produced IL-6 in response to LPS and MSCs effect *in vivo* was accompanied by additional stimulation of both micro- and macroglia. Xenogeneic (human) MSCs were almost as efficient as allogeneic (mouse) ones and regular injections of human MSC-conditioned medium also produced positive effect. These data allow suggesting MSCs as a potential therapeutic tool to cure neuroinflammation-related cognitive pathology.

## Introduction

Alzheimer disease (AD) is an age-dependent neurodegenerative disorder resulting in impairment of memory, speech, and practical habits. The brains of AD patients are characterized by cholinergic deficiency and accumulation of extracellular senile plaques formed by oligomerized amyloid beta (Aβ) peptides. AD is accompanied by inflammatory reactions; moreover, neuroinflammation often precedes the development of cognitive symptoms and may be regarded as one of the pathogenic factors causing neurodegeneration (reviewed in Skok and Lykhmus, 2016).

Cholinergic deficiency is manifested as the decrease of acetylcholine content in the brain due to decreased activity of choline acetyltransferase and the loss of nicotinic acetylcholine receptors (nAChRs) in the cognitively important brain areas leading to degeneration of cholinergic neurons (Keverne and Ray, 2005). Consequently, the use of either acetylcholine esterase (AChE) inhibitors or selective agonists of certain nAChR subtypes aimed to enhance cholinergic signaling has been suggested as a current medication for symptomatic treatment of AD patients (Russo et al., 2014).

The nAChRs are ligand-gated ion channels composed of various combinations of α (α1–α10) and β (β1–β4) subunits (reviewed in Changeux, 2012). The two main nAChR subtypes found in the brain are α7 and α4β2 (Champtiaux and Changeux, 2002; Dineley et al., 2015) and both of them are related to AD pathogenesis (Wevers et al., 1999; Guan et al., 2000; Gotti et al., 2006; Posadas et al., 2013). The α4β2 nAChR signaling underlies the pro-cognitive effects of nicotine (Picciotto et al., 2001), the absence of α4β2 nAChRs in knockout mice favors neurodegeneration upon ageing (Zoli et al., 1999), and the density of α4β2 nAChRs is decreased in people with neurodegenerative diseases including the AD (Wevers et al., 1999; Guan et al., 2000). The α7 nAChRs directly interact with Aβ to affect its proper metabolism (Wang et al., 2000; Parri and Dineley, 2010). This nAChR subtype is expressed in both the plasma membrane and mitochondria of the brain cells to support their viability (Parada et al., 2010; Gergalova et al., 2012; Lykhmus et al., 2014). In addition, the α7 nAChRs expressed in the glial cells regulate inflammatory reactions in the brain (Suzuki et al., 2006; Tyagi et al., 2010; Thomsen and Mikkelsen, 2012).

In our previous studies, we showed that mice regularly injected with bacterial lipopolysaccharide (LPS) possessed decreased levels of α7 and α4β2 nAChRs, elevated amounts of Aβ (1–42) in the brain, and demonstrated episodic memory decline (Lykhmus et al., 2015b, 2017). The LPS treatment stimulated astrocytosis in the cortex and striatum and evidently decreased the number of cells in the hippocampus and striatum. The brain mitochondria of LPS-treated mice also contained less α7 nAChRs, more Aβ peptides (1–40) and (1–42) and released more cytochrome *c* (cyt *c*) in response to apoptogenic doses of Ca^2+^. These data allowed us to suggest that neuroinflammation caused by external inflammatory stimuli led to α7 nAChR down-regulation, accumulation of Aβ (1–42), and mitochondria impairment resulting in memory decline and finally neurodegeneration.

Mesenchymal stem cells (MSCs) are self-renewing multipotent cells able to differentiate into multiple cell types including neurons (NIH Stem Cell Information Home Page, 2016). In addition, MSCs produce numerous trophic and growth factors affecting neurogenesis, synaptogenesis, astrocytosis and cell survival (Konala et al., 2016). The efficiency of regenerative MSC therapy has been studied in many experimental models (Kariminekoo et al., 2016) including transgenic mice bearing AD-related mutations (Yang et al., 2013; Chang et al., 2014; Shin et al., 2014) and their role in modulating inflammation (Klinker and Wei, 2015; Zachar et al., 2016) have been demonstrated. In addition, the regenerative potential of MSC-secreted factors vs. the cells *per se* is being widely discussed (Konala et al., 2016).

In the present study, we put an aim to investigate whether pathogenic effect of LPS on the brain and behavior of mice can be prevented or reversed by MSCs, and if yes, whether the effect can be reproduced by MSC-produced soluble factors.

## Materials and Methods

### Materials

All reagents were of chemical grade and purchased from Sigma-Aldrich (Saint Louis, USA), unless specially indicated. Antibodies against α3(181–192), α4(181–192), α7(179–190), α9(11–23), β2(190–200) or β4(190–200) nAChR fragments and rabbit cyt *c*-specific antibodies were generated using methods previously developed in our lab (Skok et al., 1999; Koval et al., 2004, 2011; Lykhmus et al., 2010; Gergalova et al., 2014). The antibodies were biotinylated according to standard procedures (Harlow and Lane, 1988). Antibodies against Aβ (1–42), ionized calcium binding adaptor molecule 1 [(Iba-1) or allograft inflammatory factor (AIF-1)] and neutravidin-peroxidase conjugate were purchased from ALT Ukraine Ltd (representing Thermo Fisher Scientific in Ukraine). Rabbit polyclonal antibody against glial fibrillary acidic protein (GFAP) was from Dako (Agilent Technologies); goat anti-rabbit IgG Alexa 488-labeled and IL-6-specific antibody pair were from Invitrogen.

### Animals

As MSCs recipients, we used C57BL/6 J female mice 3–5 months of age. Placental MSCs were obtained from 6 week-old female FVB wild-type pregnant mice, and GFP-labeled MSCs were obtained from FVB-Cg-Tg (GFPU) 5Nagy/J mice, transgenic by green fluorescent protein (GFP) gene. Mice were kept in the animal facilities of either Palladin Institute of Biochemistry NAS of Ukraine or Dmitry F. Chebotarev Institute of Gerontology NAMS of Ukraine in Kyiv. Mice were housed in quiet, temperature-controlled rooms, and provided with water and food pellets *ad libitum*. Before removing the brains, mice were sacrificed by cervical dislocation. All procedures complied with the ARRIVE guidelines, were carried out in accordance with the Directive 2010/63/EU for animal experiments and were approved by the Animal Care and Use Committee of Palladin Institute of Biochemistry.

### Mesenchymal Stem Cells Isolation, Propagation, and Characterization

Human MSCs (hMSCs) were obtained from Wharton jelly (WJ) using the explant method (Maslova et al., 2013; Shuvalova and Kordium, 2016). Umbilical cords were collected from healthy donors (39–40 weeks of gestation) after their consent. The umbilical cord fragment (5–10 cm) was washed with PBS, the vessels were mechanically removed. WJ was cut into 0.4–0.5 mm pieces that were placed in the 75 cm^2^ cultural flasks containing complete growth medium α-MEM (BioWest, Austria) supplemented with 10% fetal bovine serum (Invitogen), penicillin 100 U/ml (Arterium, Ukraine), and streptomycin 100 μg/ml (Arterium, Ukraine). Cultivation was performed under conditions of humidified air with 5% CO_2_ at 37°C. The medium was changed every 4–5 days. The first attached cells were visible on the 7–10th day. After 14 days the clones reached the size and confluence (70–80%) sufficient for passing, which was performed by standard method with the use of trypsin-EDTA mixture (Shuvalova et al., 2013). The surface marker proteins CD34, CD45, CD90, CD73, CD105 expression was determined at the second passage by flow cytometry (BD FACS Aria) with FITC- and PE- conjugated antibodies (UsBiological, USA) according to minimal criteria for defining multipotent mesenchymal stromal cells (Dominici et al., 2006). The cells of the second passage were used for both *in vitro* assays and transplantation into LPS-treated mice.

Murine placental multipotent mesenchymal stem cells (mMMSCs, further mMSCs) were obtained from FVB-Cg-Tg (GFPU) 5Nagy/J mice 19th day of pregnancy. Under sterile conditions, placentae were transferred into a Petri dish with cold PBS, containing antibiotics. Fetal membranes were minced and incubated with 0.1% collagenase type I (Sigma-Aldrich, USA) for 90 min at 37°C. Cell pellet obtained after digestion and filtration was washed and seeded in 75 cm^2^ flasks containing culture medium DMEM-LG (Low Glucose, 1 g/L) supplemented with 10% fetal bovine serum, penicillin 100 U/ml, streptomycin 100 μg/ml and 1:100 nonessential amino acids (Sigma-Aldrich, USA). Cultivation was carried out in CO_2_-incubator under conditions of humidified air with 5% CO_2_ at 37°C. The medium was changed every 3–4 days. After approximately 14 days, the cells were rinsed with Dulbecco’s Phosphate Buffered Saline (Sigma-Aldrich, USA), and then exposed to pre-warmed trypsin–EDTA (0.25% trypsin, 4 mM EDTA, Invitrogen) for 2 min. The resulting detached cells were resuspended in serum-supplemented medium, counted and seeded as first passage cultures at 4,000 cells per cm^2^. Subcultivation was performed at 80% confluence of the monolayer. Cells of the second passage were used in the experiment (Fhilho and Oliveira, 2012).

Phenotyping of cells for markers CD34, CD44, CD45, CD73, CD90, CD105 was performed using fluorochrome-labeled monoclonal antibodies to mouse membrane antigens by flow cytometry. Obtained cell cultures satisfied criteria of MMSCs by phenotype and ability to directed multilinear differentiation.

### *In vitro* Assays

Mouse MSCs (4 × 10^4^ cells per well) were seeded in 96-well tissue culture plates containing complete growth medium DMEM/F12 supplemented with 10% fetal bovine serum, penicillin 100 U/ml, streptomycin 100 μg/ml (all–Sigma-Aldrich, USA) and were cultured in the presence of different doses of LPS at 37°C and 5% CO_2_ during 72 h. Then, the cell supernatant was collected and the cells’ quantity/viability was studied in MTT test (Carmichael et al., 1987). The supernatants were tested for the presence of IL-6 using the Murine IL-6 ELI-Pair kit from Diaclone (Gen-Probe, France), according to manufacturer’s instructions.

### Animal Treatment and Brain Preparations

In the first set of experiments, three groups of C57Bl/6 mice, eight animals per group, were intraperitoneally injected with 2 mg kg^−1^ LPS (*E. coli* strain 055:B5) in 0.1 ml of saline. Two of these groups, in addition, obtained intravenously, in the tail vein, 10^6^ mMSCs or hMSCs in 0.1 ml of incubation medium. Three weeks thereafter, mice were examined in behavioral novel object recognition test, sacrificed and their brains were removed for examination.

In the second set of experiments, three groups of mice, five animals in each, were injected with LPS as described above. After 3 weeks, the mice were examined in behavioral test and one group obtained hMSCs (10^6^ in the tail vein), while another group was injected intraperitoneally with 0.3 ml of hMSC-conditioned medium obtained after 2 days of cells incubation in serum-free medium. Injections of conditioned medium were repeated every 7 days for 3 weeks more and mice were examined in behavioral test every week. In a month, a week after the last conditioned medium injection, mice were sacrificed and their brains were removed for examination.

To study if intravenously introduced MSC penetrate into the brain parenchyma, GFP-labeled mMSCs (10^6^ per animal) were injected into two mice pre-injected with LPS a day before. Mice were sacrificed 24 and 72 h thereafter and their brains were removed for examination.

For sandwich ELISA experiments and mitochondria examination, the mouse brains were homogenized in a glass homogenizer. The homogenate was fractionated into mitochondria and non-mitochondria by standard procedure of differential centrifugation (Gergalova et al., 2012; Lykhmus et al., 2015a). The purity of fractions obtained was evaluated by ELISA using the antibodies against different cellular compartments, as described previously (Uspenska et al., 2017). Live mitochondria were further examined in functional test of cytochrome c (cyt *c*) release (see below), while the pellets of both mitochondria and non-mitochondria fractions were used to prepare the detergent lysates, as described previously (Lykhmus et al., 2015b). Protein concentration was measured with the BCA kit (Thermo Scientific, France).

For immunohistochemistry studies, the brains were fixed in 4% paraformaldehyde for 48 h, washed in PBS, dehydrated with increasing concentrations of alcohol and embedded in ParaplastX-TRA (McCormick Scientific LLC). The paraplast-embedded specimens were cut into serial frontal 5 μM sections with rotational microtome (HM 325, MICROM International GmbH). The sections were placed onto adhesive microscopic slides to be further examined by immunohistochemistry.

To study MSC penetration into the brain, the brains were fixed in 4% paraformaldehyde and cut by vibratome into coronal 40 μm sections. The floated sections were placed onto microscopic slides to be examined by confocal microscopy.

### Elisa Assays

To determine the level of various nAChR subunits within the brain or mitochondria preparations, the immunoplates (Nunc, Maxisorp) were coated with rabbit α7(1–208)-specific antibody (20 μg/ml), blocked with 1% BSA, and the detergent lysates of brain tissue or mitochondria were applied into the wells (1 μg of protein per 0.05 ml per well) for 2 h at 37°C. The plates were washed with water and the second biotinylated α3(181–192)-, α4(181–192)-, α7(179–190)-, α9(11–23)-, β2(190–200)- or β4(190–200)-specific antibody was applied for additional 2 h being revealed with Neutravidin-peroxidase conjugate and *o*-phenylendiamine-containing substrate solution.

To determine the level of Aβ (1–42) bound to α7 nAChR, the plates were coated with α7(1-208)-specific antibody, and the α7-Aβ complex from the brain or mitochondria preparation applied as described above was revealed with biotinylated Aβ (1–42)-specific antibody, Neutravidin-peroxidase conjugate and *o*-phenylendiamine-containing substrate solution. The optical density was read at 490 nm using Stat-Fax 2000 ELISA Reader (Awareness Technologies, USA).

### Immunohistochemistry and Confocal Microscopy

Before immunohistochemical staining, the brain sections were de-paraffinated by standard procedure; the nonspecific binding was blocked with 1% BSA in PBS (30 min, RT). For staining the Aβ (1–42), the slides were incubated with biotinylated mouse Aβ (1–42)-specific antibody (1:200) overnight at room temperature, washed with PBS and incubated with Extravidin-Cy3 (1,200) in 1% BSA-containing PBS for 1 h at RT. Nonspecific binding was blocked with goat anti-mouse IgG.

For staining the astrocytes, the slides were incubated with rabbit anti-GFAP antibody (1:100) followed by goat anti-rabbit-Alexa 488.

For staining the microglia, the slides were incubated with biotinylated anti-Iba-1 antibody (1:100) followed by Streptavidin-Cy3.

As negative controls, the incubations without primary antibody were performed in both control and experimental sections.

All slides with paraplast sections were embedded in MOWIOL-DABCO, while slides with floated brain sections were embedded in Vectashield and examined under Zeiss LSM 510 Meta confocal lasers canning microscope (Zeiss, Germany). The brain regions were identified according to Paxinos and Franklin, 2001.

### Mitochondria Functional Assay

The purified live mitochondria (120 μg of protein per ml) were incubated with either 0.1 μM CaCl_2_ or 0.9 μM CaCl_2_ for 5 min at room temperature and were immediately pelleted by centrifugation (10 min, 7,000 g) at 4°C. The incubation medium contained 10 mM HEPES, 125 mM KCl, 25 mM NaCl, 5 mM sodium succinate and 0.1 mM Pi(K), pH 7.4. The mitochondria supernatants were collected and tested for the presence of cyt *c* by sandwich assay as described previously (Gergalova et al., 2012, 2014).

### Behavioral Experiments

Mice of all groups were tested in the “Novel Object Recognition” (NOR) behavioral test (Antunes and Biala, 2012; Lykhmus et al., 2015a,b) prior and post-treatments. Briefly, the animals were individually placed in a rectangular novel open field containing two identical objects with distinctive features (shape and texture). The animals were subjected to a 10-min session of exploration of the objects followed by 10 min in a waiting cage. During the second 10-min session, one of the objects was replaced by a novel one, and we scored the time spent in contact with each object. It is widely acknowledged that rodents spontaneously explore novel objects by touching the objects with their nose and prefer novel objects to familiar ones that reflect their episodic memory (Save et al., 1992; Thinus-Blanc, 1996). Therefore, the time spent in contact with each object reflects the time of exploration for the object. The results of NOR test are presented as discrimination index (DI) calculated as the difference in the number of “novel” and “famous” object explorations divided by the total number of explorations of two identical objects.

### Statistical Analysis

ELISA experiments have been performed in triplicates and mean values for individual mice were used for statistical analysis assessed using one-way ANOVA test. Behavioral tests were also performed in triplicate for each mouse and mean values for individual mice were taken for statistical analysis. The data are presented as mean ± SD; *p < 0.05; ** *p* < 0.005; *** *p* < 0.0005.

## Results

### Both Mouse and Human MSCs Prevent Pathogenic Effect of LPS on Mouse Brains and Behavior

**Figure 1:**
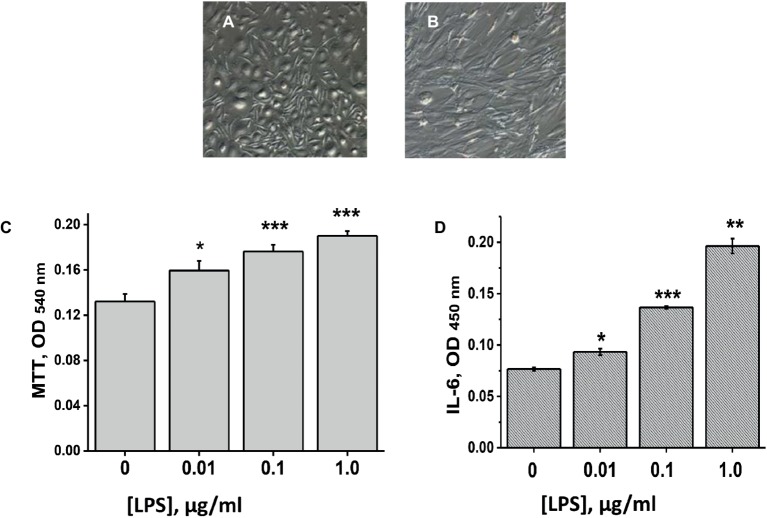
Placental mMSCs cultured *in vitro*. **(A,B)** – microscopic images of primary **(A)** and passage 2 cells **(B)**; their proliferation (MTT test) **(C**), and IL-6 production **(D)** under the effect of LPS. Each column in C and D corresponds to M ± SD of triplicate measurements; *p < 0.05; **p < 0.005; ***p < 0.0005 compared to Ctrl (no LPS).

We used MSCs from two sources: either human umbilical cord (hMSCs) or mouse placenta (mMSCs). Both types of cells satisfied minimal criteria for defining multipotent mesenchymal stromal cells according to CD34, CD44, CD45, CD90, CD73, CD105 surface markers expression (Dominici et al., 2006) and were used for transplantation after two passages *in vitro*. Morphology of mMSCs in primary culture and at the second passage is shown in Figures 1A,B. Both mMSCs and hMSCs were shown to proliferate and to produce IL-6 in response to LPS stimulation (data for mMSCs are shown in Figures 1C,D).

In the first set of experiments, mice were injected intraperitoneally with LPS and intravenously with either hMSCs or mMSCs (10^6^ cells per mouse) at the same time. Three weeks later, they were examined in memory test and their brains and brain mitochondria were studied for the levels of α7 nAChR, Aβ (1–42) and cyt *c* released upon Ca^2+^ stimulation.

**Figure 2:**
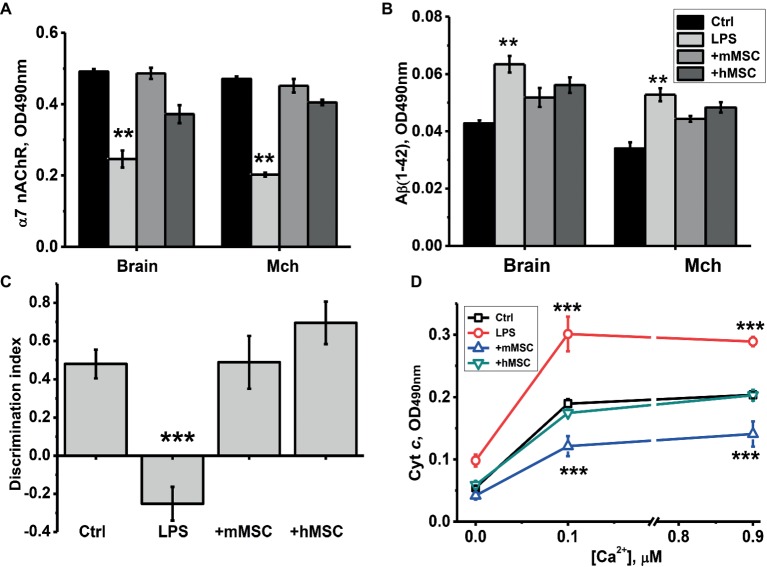
The effect of either hMSCs or mMSCs on α7 nAChR **(A)** or Aβ (1–42) **(B)** levels in the brain, memory impairment **(C)** and cyt *c* release from the brain mitochondria (Mch) under the effect of Ca^2+^ **(D)**. Ctrl – samples of non-treated mice. Each column **(A–C)** or point **(D)** corresponds to M ± SD of data for separate mice in each group (*n* = 8). **p < 0.005; ***p < 0.0005 compared to Ctrl. Designations of columns are similar in **(A)** and **(B)**.

It was found that MSCs injection prevented α7 nAChR decrease, Aβ (1–42) accumulation and increased cyt *c* release from mitochondria, as well as episodic memory decline caused by LPS. Human MSCs were almost as efficient as mouse ones in a majority of tests (Figure 2).

**Figure 3:**
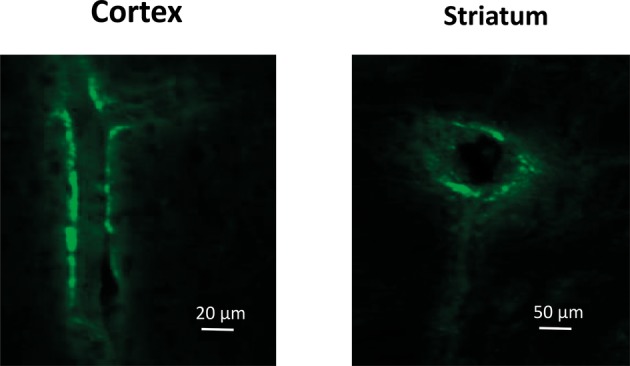
Confocal microscopy images of the blood vessels in the *Cortex (*lengthwise projection*)* and *Striatum* (cross-cut projection) of LPS-treated mice prepared 72 h after intravenous injection of GFP-labeled mMSCs.

The use of GFP-labeled mouse MSCs demonstrated that green fluorescent signal could be found along/around the brain blood vessels 3 days after intravenous injection (Figure 3).

### Both Human MSCs and Their Conditioned Media Reverse the Pathogenic Effects Developed 3 Weeks After LPS Injection

The second set of experiments was undertaken to find out whether the MSCs effect is due to cellular or humoral influence and whether it is only prophylactic or can also be therapeutic, i.e., cure the already developed pathogenic symptoms caused by LPS.

**Figure 4:**
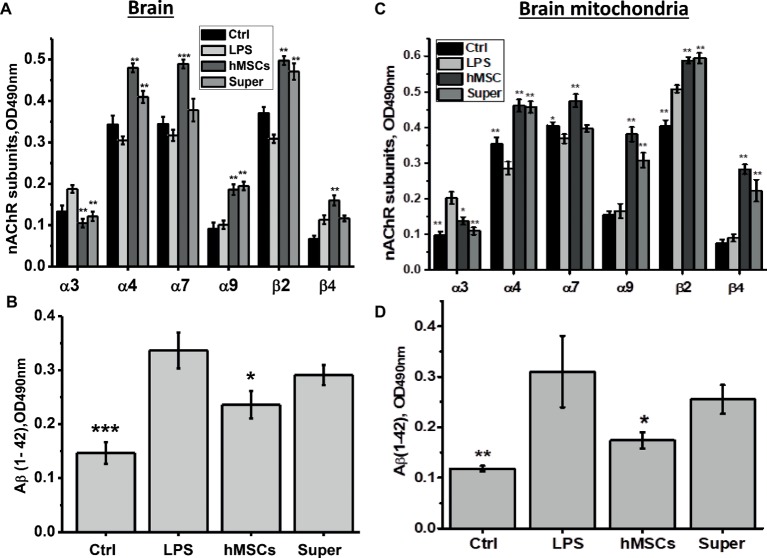
The effects of either hMSCs or their conditioned medium (Super) on the level of nAChR subunits **(A,C)** or α7-bound Aβ (1–42) **(B,D)** in the brain and brain mitochondria of mice pre-injected with LPS 3 weeks before. Each column corresponds to M ± SD of data for separate mice in each group (*n* = 5) *p < 0.05; **p < 0.005; ***p < 0.0005 compared to LPS-treated samples/mice.

**Figure 5:**
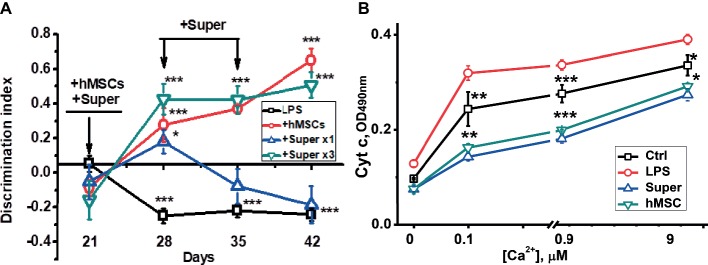
The effects of either hMSCs or their conditioned medium (Super) on episodic memory **(A)** and cyt *c* release from the brain mitochondria under the effect of Ca^2+^ **(B)** in mice pre-injected with LPS 3 weeks before. Arrows in **(A)** indicate the time points of LPS, MSCs or conditioned medium injections. Each point corresponds to M ± SD of data for separate mice in each group (*n* = 5) *p < 0.05; **p < 0.005; ***p < 0.0005 compared to LPS-treated samples/mice.

For this purpose, mice were injected intravenously with xenogeneic (human) MSCs 3 weeks after LPS injection when memory decline was already observed. Another group of LPS-treated mice was injected once per week intraperitoneally with the conditioned culture medium in which hMSCs were grown to 80% confluency and were maintained for 2 days without serum. Mice were examined in memory test every week thereafter, then sacrificed and their brains and brain mitochondria were studied as in the previous set of experiments. In this case, we studied a broader range of nAChR subunits in both the mitochondrial and nonmitochondrial brain fractions. It was found that either hMSCs or their supernatants up-regulated α4, α9 and β2 nAChR subunits in their brains (Figure 4A) and brain mitochondria (Figure 4C); MSCs additionally up-regulated β4 subunits and decreased the level of Aβ (1–42) (Figures 4B,D). Either hMSCs or their supernatants also restored memory of LPS-treated mice and significantly improved mitochondria sustainability to Ca^2+^ (Figures 5A,B). In contrast to MSCs, which supported memory of LPS-treated mice for at least 3 weeks, the effect of a single injection of conditioned medium was transient and disappeared after 2 weeks (Figure 5A).

**Figure 6:**
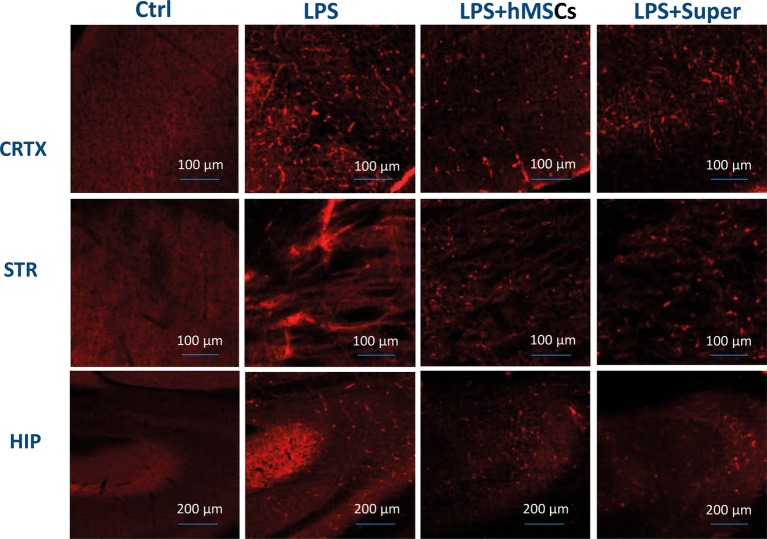
Confocal microscopy images of the brain slices obtained from non-treated (Ctrl), LPS-treated, LPS + hMSCs-treated or LPS + Sup-treated mice and stained with Aβ (1–42)-specific antibody (red). CRTX, cortex; STR, striatum; HIP, hippocampus.

The visible decrease of Aβ (1–42) accumulated in the frontal cortex, striatum and hippocampus of LPS injected mice under the effect of either MSC or their conditioned medium was found by means of immunohistochemistry (Figure 6).

**Figure 7:**
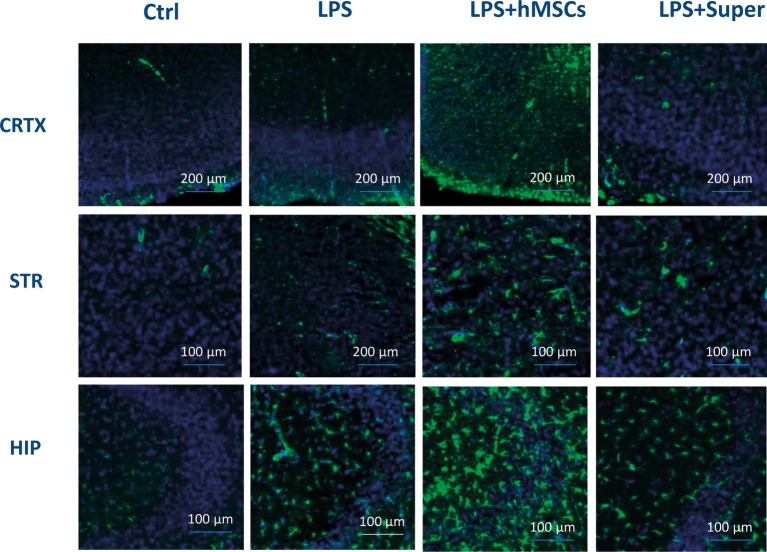
Confocal microscopy images of the brain slices obtained from non-treated (Ctrl), LPS-treated, LPS + hMSCs-treated or LPS + Sup-treated mice and stained with GFAP-specific antibody (green) and DAPI (blue). CRTX, cortex; STR, striatum; HIP, hippocampus.

**Figure 8:**
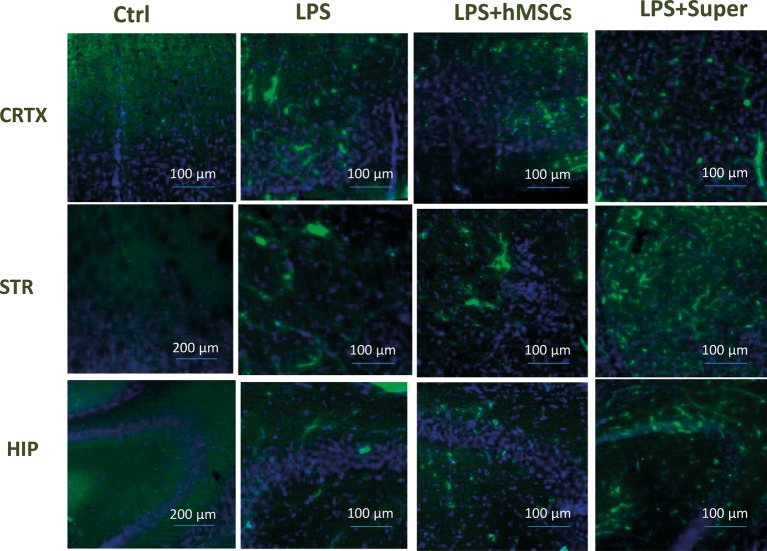
Confocal microscopy images of the brain slices obtained from non-treated (Ctrl), LPS-treated, LPS + hMSCs-treated or LPS + Sup-treated mice and stained with Iba-1-specific antibody (green) and DAPI (blue). CRTX, cortex; STR, striatum; HIP, hippocampus.

Finally, MSC visibly increased the green signal for GFAP (Figure 7), while their conditioned medium increased Iba-1-specific staining (Figure 8) in the brains of LPS-treated mice. Although no quantitative analysis has been performed in this experiment, the data allow suggesting that the treatments increased the number of activated astrocytes (GFAP) or microglial cells (Iba-1).

## Discussion

The data obtained in the first set of experiments indicated that MSCs injected intravenously prevent the pathogenic effect of LPS on the brain, brain mitochondria and behavior of LPS-treated mice. In particular, they prevented the α7 nAChR decrease, Aβ (1–42) accumulation, mitochondria and episodic memory impairment caused by LPS injection. Xenogeneic (human) MSCs were almost as efficient as allogeneic (mouse) ones suggesting that their effect was mostly due to soluble growth/trophic factors produced. This suggestion was approved in the second set of experiments where regular injections of hMSC-conditioned medium appeared to be almost as efficient as MSC themselves. In addition, it was found that MSCs can not only prevent the pathogenic effect of LPS, but also reverse the already developed nAChR decrease, Aβ (1–42) accumulation, mitochondria, and memory impairment. The anti-LPS effect of MSCs was accompanied by additional stimulation of both micro- and macroglia suggesting that these cells are the targets of MSC-produced soluble factors. We can speculate that positive effect of MSCs is, at least partly, due to activation of trophic functions of glial cells.

The use of multipotent stem cells is an attractive idea of regenerative medicine, in particular, for neurodegenerative disorders like AD. The therapeutic effect of MSCs has been already studied in several AD models. The first studies were performed with the bone-marrow-derived MSCs transplanted intracerebrally (Lee et al., 2009, 2010a,b, 2012; Zhang et al., 2012; Bae et al., 2013) in mice either pre-injected with Aβ (Lee et al., 2009, 2010a) or transgenic for APP/PS1 (Lee et al., 2010b, 2012; Bae et al., 2013). A big piece of studies was performed with human MSCs from adipose tissue, a good source of autologous MSCs (Ma et al., 2013; Chang et al., 2014; Yan et al., 2014). Human umbilical cord MSCs were also transplanted intracerebrally in APP/PS1 mice (Lee et al., 2012; Yang et al., 2013). In all cases, the improvement of cognitive (memory) and synaptic functions, as well as reduced Aβ deposition has been observed that is in accord with our data. Advantage of our model is that the Aβ accumulation in the brain occurred in a natural way in genetically non-modified mice. The wild-type mice do not form senile plaques; however, significant accumulation of soluble Aβ could be observed and it was significantly decreased upon MSC injection. Moreover, we show that MSCs can not only prevent the pathogenic effect of LPS, but cure the already developed pathogenic symptoms including memory impairment.

In contrast to the studies cited above, we injected MSCs intravenously. The brain is protected from penetration of peripheral cells by the blood-brain barrier. However, the integrity of the cerebral vasculature is compromised following inflammation (Zlokovic, 2008). In particular, a diminished function of the blood-brain barrier is an early event in multiple sclerosis when inflammation facilitates the massive influx of leukocytes into the brain parenchyma inducing demyelination, tissue damage and axonal disfunction (Kamphuis et al., 2015). Previously, we reported that the blood-brain barrier in LPS-treated mice had been disturbed to allow the nAChR-specific antibody penetration (Lykhmus et al., 2015b). Here, we show that MSCs can be found along the brain vessels of LPS-pre-treated mice 3 days after intravenous injection. This is in accord with the data of Lee et al., who found intraarterially injected MSCs in the brain vasculature (Lee et al., 2016). It was also shown that, at least *in vitro*, MSCs possess leukocyte-like molecular mechanisms enabling interaction with vascular endothelial cells (Rüster et al., 2006). Therefore, it is reasonable to expect that MSCs can penetrate the brain parenchyma *via* transmigration. Indeed, intravenously injected mouse bone marrow cells or human adipose-derived MSCs did cross the blood-brain barrier and migrated into the brain in a rat (Salem et al., 2014) or mouse models for AD (Kim et al., 2012). The AD pathology in humans is accompanied by elevated pro-inflammatory cytokines, which can affect the integrity of the blood-brain barrier (Zenaro et al., 2017; Toropova et al., 2018). However, whether its damage is comparable to that found upon multiple sclerosis and other autoimmune pathologies of the central nervous system and whether it is sufficient to allow intravenously injected MSC to penetrate the brain parenchyma is a reasonable question which needs to be addressed.

The important fact, not examined in previous studies, is that MSC injection restores/elevates the level of nAChRs in the brain and brain mitochondria decreased as a result of LPS treatment. The MSCs up-regulated α4, α7, α9, β2 and β4 subunits in the brain and brain mitochondria, while the conditioned medium did not affect α7 subunits decreased by LPS. Both α7 and α4β2 nAChRs expressed in the brain are involved in regulating cognition and memory (Gotti et al., 2006) and mitochondria-expressed α7β2, α4β2, and α9 nAChRs are involved in the anti-apoptotic pathways (Gergalova et al., 2012, 2014; Lykhmus et al., 2014; Uspenska et al., 2018); therefore, their increased levels make mitochondria more resistant to apoptogenic influence and support the viability of brain cells. In one of the published papers, MSCs were introduced together with galantamine-containing nanoparticles; that was expected to inhibit AChE and increase cholinergic signaling (Misra et al., 2016). Our data indicate that MSCs themselves contribute to cholinergic signaling in the brain by increasing the level of nAChRs.

Another important question arising from positive effects of MSC in AD models is whether they are due to direct involvement of differentiated MSC into neuronal networks in the brain (the true regenerative medicine) or are mediated by numerous trophic and growth factors produced by MSCs to stimulate the host cells. The latter possibility is therapeutically important because it allows avoiding cell transplantation by substituting it with the use of MSC-produced substances. Previous studies demonstrated that human MSCs stimulated neurogenesis both *in vitro* (Park et al., 2016) and *in vivo* (Yan et al., 2014; Kim et al., 2015; Oh et al., 2015) by producing soluble factors like activin A, growth differentiation factor-15 and activating Wnt signaling pathway in neuronal progenitor cells. Moreover, it was found that MSCs produce extracellular vesicles (exosomes), which contain neprilysin, enzyme involved in Aβ degradation (Katsuda et al., 2013), and these exosomes can be suggested for AD therapy (Katsuda et al., 2015). We show here that supernatants of hMSC culture, applied intraperitoneally, improved memory of LPS-treated mice and affected their brains almost similarly to MSCs. However, in contrast to MSCs, which improved the state of mice for at least 3 weeks after a single injection, the effect of MSC supernatant was transient and regular injections were required to maintain the improvement achieved. Additional studies are required to establish whether MSC-conditioned medium can provide a stable therapeutic effect.

Finally, it is not definitely clear which cells in the brain are the targets for MSC-produced factors. The effects of MSC co-culture with neuronal progenitor cells suggested the direct influence on the brain neurons (Oh et al., 2015; Park et al., 2016). However, other studies demonstrated that intracerebrally transplanted human adipose MSCs activate microglia around senile plaques in the brain of APP/PS1 transgenic mice (Ma et al., 2013). We observed a visible activation of both microglia and astrocytes in the brains of MSC-injected mice, additional to that induced by LPS, and showed that MSCs produce IL-6 in response to LPS stimulation *in vitro*. Therefore, injecting MSCs simultaneously with LPS could stimulate MSCs for IL-6 production. IL-6 is a pro-inflammatory cytokine, but is also known as a neurotrophic factor (Hirota et al., 1996; Wagner, 1996). Recently, it was reported that mesenchymal progenitor cells derived from induced pluripotent stem cells enhance neuritogenesis *via* neurotrophin and cytokine (including IL-6) production (Brick et al., 2018). Therefore, IL-6 may be one of neurotrophic soluble factors produced by MSCs penetrating the brain that can affect both neurons and glial cells.

The data obtained put a wider question on what happens in the mouse brain under the effect of injected MSCs. The ability of MSCs to prevent LPS pathogenic effect (simultaneous injection of LPS and MSCs) indicates that MSCs do not allow such effect to develop. However, positive MSCs effect when pathological symptoms have already developed (MSCs injection 3 weeks after LPS) allows suggesting that MSCs, or their soluble factors, directly or indirectly (by activating glial cells) restore the activity of damaged brain neurons. Further experiments are needed to reveal whether the recovery observed under the MSCs effect is long-lasting or just temporal.

## Conclusions

1. Intravenously injected MSCs penetrate the brain of LPS-treated mice.
2. Either allogenic (mouse) or xenogeneic (human) MSCs prevent and reverse the pathogenic effect of LPS on the brain nAChRs, Aβ accumulation, mitochondria and memory impairment.
3. The MSCs therapeutic action is largely due to their humoral factors and is accompanied by activation of micro- and macroglia.

## Ethics Statement

This study was carried out in accordance with the recommendation of the guidelines of the Animal Care and Use Committee of Palladin Institute of Biochemistry, Kiev. The protocol was approved by the IACUC of Palladin Institute of Biochemistry.

## Author Contributions

MS, OD, SK, OL, and VKo made substantial contributions to the conception or design of the work. LK, OL, LV, KU, MS, NS, and AU contributed to acquisition, analysis, and interpretation of data for the work. MS drafted the work. OD, VKy, and AU revised it critically for important intellectual content. LK, OL, LV, KU, SK, VKo, OD, NS, VKy, and AU finally approved the version to be published. LK, OL, LV, KU, SK, VKo, OD, NS, VKy, and AU agreed to be accountable for all aspects of the work in ensuring that questions related to the accuracy or integrity of any part of the work are appropriately investigated and resolved.

### Conflict of Interest Statement

The authors declare that the research was conducted in the absence of any commercial or financial relationships that could be construed as a potential conflict of interest.

## Abbreviations

AD: Alzheimer disease
cyt *c*: cytochrome *c*
GFP: green fluorescence protein
LPS: lipopolysaccharide
MSCs: mesenchymal stem cells
nAChR: nicotinic acetylcholine receptor.
